# Mesenchymal stem cells as all-round supporters in a normal and neoplastic microenvironment

**DOI:** 10.1186/1478-811X-10-26

**Published:** 2012-09-03

**Authors:** Ralf Hass, Anna Otte

**Affiliations:** 1 Biochemistry and Tumor Biology Lab, Gynecology Research Unit, Department of Obstetrics and Gynecology (OE 6410), Medical University Hannover, Carl-Neuberg-Str. 1, 30625 Hannover, Germany

**Keywords:** Mesenchymal stem cells, Tumor cells, Microenvironment

## Abstract

Mesenchymal stem cells (MSC) represent a heterogeneous population exhibiting stem cell-like properties which are distributed almost ubiquitously among perivascular niches of various human tissues and organs. Organismal requirements such as tissue damage determine interdisciplinary functions of resident MSC including self-renewal, migration and differentiation, whereby MSC support local tissue repair, angiogenesis and concomitant immunomodulation. However, growth of tumor cells and invasion also causes local tissue damage and injury which subsequently activates repair mechanisms and consequently, attracts MSC. Thereby, MSC exhibit a tissue-specific functional biodiversity which is mediated by direct cell-to-cell communication via adhesion molecule signaling and by a tightly regulated exchange of a multifactorial panel of cytokines, exosomes, and micro RNAs. Such interactions determine either tumor-promoting or tumor-inhibitory support by MSC. Moreover, fusion with necrotic/apoptotic tumor cell bodies contributes to re-program MSC into an aberrant phenotype also suggesting that tumor tissue in general represents different types of neoplastic cell populations including tumor-associated stem cell-like cells. The present work summarizes some functional characteristics and biodiversity of MSC and highlights certain controversial interactions with normal and tumorigenic cell populations, including associated modulations within the MSC microenvironment.

## Mesenchymal stromal/stem cells represent a heterogeneous population in nearly all kinds of tissues

Mesenchymal stem cells (MSC) can be identified in and isolated from nearly all kinds of human tissues. MSC are often synonymously termed stromal cells while other reports distinguish MSC as a precursor of stromal cells. As firstly described, bone marrow-derived human MSC (BM-hMSC) still represent the most frequently investigated hMSC population to compare their properties to those of other tissue-originating hMSC populations [1]. Among these are hMSC from adipose tissue [2], peripheral blood [3], heart [4], and lung [5] but more recently, special attention was also given to hMSC obtained from a variety of different neonatal tissues. These post-natal tissues represent a useful ethically non-controversial alternative providing certain advantages as a consistent and enriched MSC source which is easily accessible. These tissues include MSC from the amniotic fluid [6], amniotic membrane [7-9], chorionic membrane [10], chorionic villi [11], decidua [10], whole placenta [12,13], cord blood [14], Wharton's jelly [15] and whole umbilical cord (UC-MSC) [16].

### Proliferative capacity of MSC

The hMSC originating from different human tissues or organs are designated as stem/stromal or stem-like cells since they share functional properties such as continuous cell cycle progression and plasticity by the capability to differentiate at least along the mesodermal lineage. However, several tissue-originating MSC display significant differences in their proliferative capacities. According to the tissue-specific microenvironment of the diverse MSC populations, UC-MSC exhibit a higher proliferation potential than BM-MSC [17-19]. Thus, the mean doubling time of the UC-MSC revealed about 24 h and remained almost constant for up to 10 cell passages. In contrast, the population doubling time of BM-MSC reached approximately 40 h and considerably increased already after 6 cell passages [20]. Likewise, adipose tissue-derived MSC also demonstrated an elevated growth rate as compared to BM-MSC [21]. Morphological evaluations revealed a larger BM-MSC size in contrast to adipose tissue-derived MSC which was also accompanied by an earlier *in vitro* senescence of BM-MSC [22]. However, the *in vitro* results of MSC may dramatically differ from the *in vivo* situation. In this context, effects of the cellular microenvironment such as oxidative stress and DNA damage clearly influence the proliferative capacity and premature aging of the cells [23]. Indeed, hMSC demonstrate enhanced proliferative potential in a hypoxic microenvironment which is also paralleled by an altered energy-consuming metabolism [24].

### Differentiation capacity and markers of MSC

With respect to differentiation capacity, previous work has demonstrated that MSC can acquire certain functions associated with adipogenic, chondrogenic or osteogenic maturation [25-27]. This differentiation potential of MSC supports local tissue-specific precursor cells of damaged organs, since the amount of these precursor cells to retrodifferentiate to a more undifferentiated phenotype is often insufficient to cope with the cell replacement requirements of the injured tissues [28,29]. Some reports suggested the capability of distinct MSC populations to differentiate along cell lineages of all three germ layers as described for amniotic fluid-derived hMSC and for a certain clonal subpopulation of decidual-derived hMSC [6,30]. However, a trans-germ line differentiation via ectodermal or endodermal lineages including maturation into hepatocytes, cardiomyocyte or neuronal phenotypes remains controversial [31]. Nevertheless, the initially tissue- or organ-derived cells harbor a heterogeneous population of mesenchymal stromal cells with stem cell-like properties since a variety of distinct subpopulations can be isolated from such an entity by centrifugal counter flow elutriation. These isolated MSC subpopulations exhibit different growth rates and aging properties by quantification of senescence-associated β-galactosidase expression [32,33]. This kind of heterogeneity may also explain differences in the activation status and alterations in cell biological properties and functions within the same hMSC population. According to this cellular diversity, a panel of multiple markers is required for the characterization of mesenchymal stem cells. Minimal criteria for all hMSC populations have been defined by the International Society for Cellular Therapy to characterize multipotent mesenchymal stem cells which include the capacity to plastic adherence, differentiation potential (at least osteogenic, chondrogenic and adipogenic) and expression of the cell surface markers CD73, CD90 and CD105 [34]. In addition to the necessity of these three surface molecules on hMSC, further criteria require the simultaneous absence of a variety of other specific markers including the monocytic CD14, the endothelial CD31, the hematopoietic stem cell marker CD34 and the lymphocyte CD45 surface antigen [3].

Although hMSC have a common minimal expression marker profile of surface receptors, additional distinct surface molecules and metabolic alterations can be detected between different hMSC populations which may determine their functional diversity within the different tissues. For example, umbilical cord-derived (UC) hMSC and some adipose tissue-derived MSC express significant levels of CD44 in contrast to bone-marrow-derived (BM) hMSC. However, there are controversial reports on CD44 expression in BM-hMSC which suggested that the original BM-hMSC represent the CD44^-^ phenotype whereby CD44 expression is acquired in the course of *in vitro* culture of BM-hMSC [32,35]. The CD44 surface molecule is also termed as hyaluronan receptor which consists of several splice variants and binds supramolecular structures of highly sulfated proteoglycans, enabling a link of several protein components in close vicinity. Certain matrix-metalloproteinases (MMPs) can associate with distinct isoforms of the transmembrane glycoprotein CD44 which anchors the proteinase to the cell surface. This CD44-MMP association mediates reorganization of distinct extracellular matrix (ECM) components or contributes to focus the proteinase activities e.g. towards growth factor activation such as cleavage of pro-heparan-binding epidermal growth factor (pro HB-EGF) for the generation of soluble active sHB-EGF [36]. A similar formation of this ternary growth factor complex is also observed in young human mammary epithelial cells in contrast to their aging counterparts and derailed signaling in this pathway can contribute to aging-associated breast cancer development [37-40]. Therefore, specific functions of CD44 appear to be important for MSC functions in the highly enriched glucose-aminoglycan environment of the umbilical cord or the adipogenic breast tissue but to lesser extend in the bone marrow.

**Figure 1:**
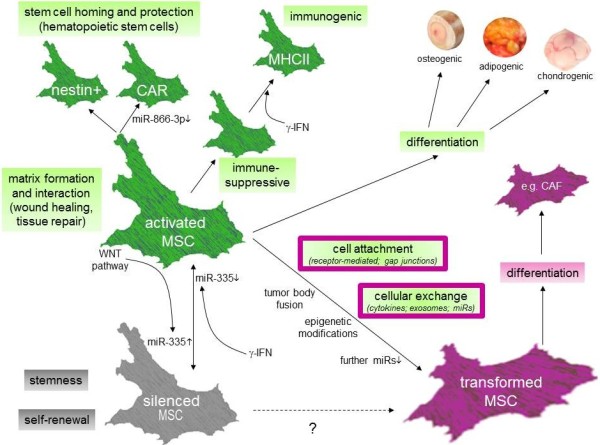
**MSC functions, activation status-dependent interaction levels of MSC and susceptibility to transform to an aberrant MSC phenotype following acquisition of tumorigenic compounds (DAMPs or apoptotic bodies).** (CAR = related stromal-derived factor-1 (SDF-1)-abundant reticular cells; CAF = cancer-associated fibroblast.

Further markers are proposed for MSC which characterize at least a certain MSC subpopulation. Such markers include Stro-1 identified in BM-MSC [41]. The Stro-1^+^ BM-MSC can inhibit lymphocyte proliferation [42], although Stro-1 expression has also been reported in a small population of CD34^+^ hematopoietic stem cells and endothelial cells [43]. Moreover, vascular cell adhesion molecule-1 (VCAM-1, CD106), a member of the immunoglobulin superfamily which binds to the leukocyte integrins VLA-4 and α4β7 as well as intercellular adhesion molecule-1 (ICAM-1) are also markers associated with MSC [44]. Furthermore, nestin has been identified in MSC which represents an intermediate filament usually expressed in neuronal (progenitor) cells. Nestin-expressing MSC are predominantly found in the bone marrow and play an important role in the maintenance of a hematopoietic stem cell (HSC) niche and control of HSC homeostasis [45] (Figure1). Whereas nestin is associated with cell division, proliferation and morphological changes, recent findings suggested that nestin-expressing BM-MSC can differentiate into neural cells upon exposure to an appropriate culture microenvironment [46]. 

Together, the minimal characteristics of surface markers still appear to be insufficient for defining the precise role of MSC and additional markers will be required for determining tissue-specificity, a selective MSC subpopulation, and the activation status of MSC.

## Interaction of mesenchymal stromal/stem cells and functional involvement in tissue repair, stem cell homing and angiogenesis

**Figure 2:**
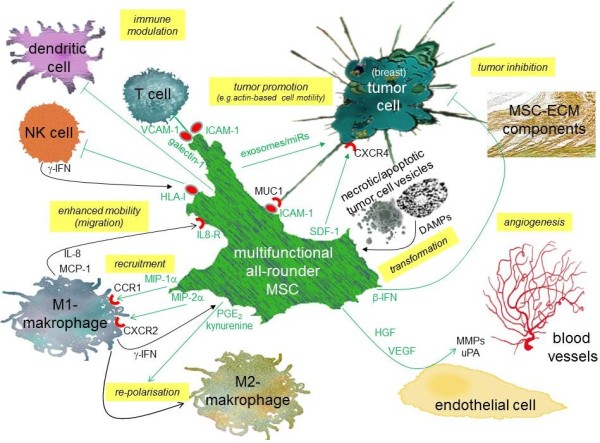
Interactions of activated MSC with surrounding immune cells (T cells, NK cells, dendritic cells, macrophages), endothelial cells with concomitant angiogenesis and neo-vascularisation, extracellular matrix (ECM) components, tumor cells (e.g. breast cancer cells) as well as damage-associated molecular patterns (DAMPs) and apoptotic bodies from necrotic/apoptotic tumor cell vesilces.

A functional heterogeneity of MSC within different tissue origins appears obvious and additionally, there are also increasing evidences for functional alterations of MSC originating from the same population [32]. These effects are associated with the special microenvironment surrounding each cell which is established by individual cell-to-cell attachments and intercellular communication processes mediated by diverse interactions with different ECM components and by the release of cytokines or the local exchange of microRNAs and membranous micro vesicles [47-49] (Figure1, Figure2). An uptake of these biological materials significantly influences regulatory pathways within the corresponding MSC metabolism. Indeed, MSC can exchange micro vesicles – also termed exosomes - which can contain certain pre-microRNAs [50]. MicroRNAs (miRs) represent small non-coding RNAs of about 20 to 22 nucleotides, which, upon sequence-specific binding to appropriate mRNAs, repress the translation of the corresponding proteins or induce a subsequent degradation of the miR/mRNA complexes. 

### Functionality of miRs in MSC

Released miR-containing exosomes into the extracellular compartment can fuse with adjacent cell membranes as a tool for intercellular communications and thereby, alter cell activities in target cells. The exchange of miRs between neighboring cells represents an integral part of MSC communication with injured cells of damaged tissue. During tissue repair and wound healing such exosomal transport allows a bidirectional exchange of miRs between injured cells and MSC. The exchange of such regulatory compounds also contributes to reprogram the phenotype of MSC to adapt to features of the damaged tissues [51]. Different miRs have been proposed to play an important role in reprogramming MSC but also in blocking differentiation pathways and regulating cell fate of MSC [52]. For instance, adipogenic differentiation was highly increased by miR105 and miR371 which was accompanied by respective gene expression changes of certain adipogenic key molecules including adiponectin and fatty acid-binding protein 4. Conversely, adipogenic differentiation of MSC can be impaired by miR-369-5p [53]. Moreover, osteogenic differentiation of MSC can be inhibited by miR-125b, miR-133, miR-135 and miR-206 which attenuate the expression of ERBB2 as one of the epidermal growth factor receptors, RUNX2 (Runt-related transcription factor 2) essential for osteoblast differentiation and skeletal morphogenesis, Smad5 as a signaling modulator of bone morphogenic proteins, and connexin-43 as a transmembrane protein and part of gap junctions, respectively. Likewise, expression of further specific miRs which contribute to the regulation of chondrogenic differentiation and pathways beyond the mesodermal lineage interfere with the corresponding functional alteration of MSC [52] (Figure1).

Moreover, miRs are also involved in directing the role of MSC in stem cells homing, particularly in keeping the homeostasis of hematopoietic stem cells. In particular, nestin-positive MSC and their related stromal-derived factor-1 (SDF-1)-abundant reticular (CAR) cells contribute to hematopoietic stem cell functions within the endo-osteal and the perivascular niche of the bone marrow. The chemokine SDF-1 (CXCL12) which is produced and released by CAR cells represents one of the key players for cell migration in several tissues and for homing of hematopoietic stem cells to the appropriate bone marrow niches [54]. The capability of CAR cells for SDF-1 production and release is strongly associated with a constitutive down-modulation of miR-886-3p in the cells since this miR specifically targets the 3' untranslated region of the SDF-1 mRNA. Furthermore, transfection of miR-886-3p into CAR cells results in the loss of SDF-1-mediated chemotaxis [55] (Figure1, Figure2).

### MSC interaction with macrophages

Further MSC activities in addition to the exchange of miRs and other exosome-containing compounds include the mutual release of chemokines and cytokines between MSC and target cells during wound healing. For the repair of damaged tissues, MSC interact with macrophages that infiltrate the wound bed in injured epithelia. During this process MSC can secrete factors such as MIP1α ((*macrophage inflammatory protein 1-alpha*), CCL3)), and MIP2α ((*macrophage inflammatory protein 2-alpha*), CXCL2, GROβ (*Growth-regulated protein beta*)) which attracts pro-inflammatory M1 macrophages via their corresponding chemokine receptors CCR1 and CXCR2, respectively, to exhibit antimicrobial activities [56,57] (Figure2). Vice versa, M1 macrophages release a panel of factors including γ-IFN, MCP-1 ((*monocyte chemotactic protein-1*), CCL2)) and IL-8 which increases the mobility of MSC [58]. To balance this inflammatory response, MSC can also repolarize these macrophages into anti-inflammatory M2 macrophages. This is mediated by γ-IFN-induced expression of cyclooxygenase in MSC followed by enhanced production and secretion of PGE_2_, whereby the MSC-mediated local increase in prostaglandin concentration contributes to silence the macrophage inflammatory activity. Similarly, indoleamine 2,3-dioxygenase which is also inducible in MSC by γ-IFN, catalyzes the conversion from tryptophan to kynurenine and tryptophan depletion in the cellular microenvironment inhibits the immune cell activity [59-61]. This re-functioning of macrophages by MSC as proposed in a model [57] further promotes wound healing (Figure2).

### MSC-induced wound healing and angiogenesis

Tissue repair and wound healing by MSC also involves angiogenesis and neo-vascularisation which requires interaction with endothelial cells. These interactions predominantly result in activation of the WNT/β-catenin and Hedgehog signaling pathways while different factors and mechanisms depend on the type of MSC tissue origin which furthermore adds to the heterogenic functionality of MSC e.g., MSC migrate to damaged tissue lesions during skin repair and replace lost cells by transdifferentiation into various skin cell types [62]. The successful maintenance of such repair mechanisms also necessitates angiogenesis and neo-vascularisation of the restored tissue. Thus, other work has demonstrated that adipose tissue-derived MSC activation of endothelial cells and subsequent vessel morphogenesis is mediated preferably via the serine proteases of the plasmin family including urokinase-type plasminogen activator in conjunction with hepatocytes growth factor and tumor necrosis factor-α (TNF-α) which contribute to vessel enlargement [63]. Whereas this activation system for vessel invasion and elongation within the fibrin-enriched extracellular matrix resembles that of fibroblasts, a different set of serine proteases such as matrix metalloproteinases are used by BM-MSC to promote angiogenesis via local endothelial cells [64]. However, chronic tissue damage such as chronic gastritis following Helicobacter pylori infection or chronic liver injuries represent organ-associated tissue defects, whereby aberrant changes in the microenvironment involving biological materials and debris from necrotic/apoptotic cells can derail MSC-mediated repair processes and may result in the promotion of neoplasias [48,49,65] (Figure1, Figure2).

In sum, the population diversity of the MSC primary cultures and the various tissue origins of the MSC populations determine their plasticity in combination with cellular interactions, released cytokines and chemokines and the multiple effects of miRs. Such a functional diversity also applies to the divergent role of MSC on interacting cell types such as immune cells.

## Interaction of MSC with immune cells for immune-modulatory and immune-privileged functions

Some important properties of hMSC are reflected by their functional interaction with a variety of immune-competent cells [66]. In particular, tissue-originating hMSC populations such as bone marrow-, adipose tissue- or umbilical cord-derived hMSC selectively alter immune cell functions by suppressing e.g. T cell activation or dendritic cells or by inhibiting the cytotoxic capacity of natural killer (NK) cells. Thus, part of the immune-suppressive capacity is attributed to a direct cell-to-cell contact between hMSC and immune cells probably through the interaction of membrane receptors, adhesion molecules or the cellular exchange of membrane vesicles. Indeed, previous work has demonstrated that activated NK cells produce and release γ-IFN which induces up-regulation of HLA class I on MSC [67]. In return, HLA molecules expressed on MSC including HLA-G increasingly bind to the inhibitory receptor ILT2 (CD85j) expressed on NK cells [68] and subsequently contribute to the suppression of NK cell functions by a down-modulation of activating NK cell receptors NKp30 (CD337), NKp44 (CD336), and NKG2D (CD314) and by inhibition of NK-cell lytic granule formation [69] (Figure1, Figure2).

Moreover, MSC express glycoproteins such as galectin-1 in intracellular compartments as well as on their cell surface which selectively inhibits T cell activation. Thus, galectin-1 knockdown in MSC partially restores the proliferation of CD4^+^ and CD8^+^ T cells without any detectable effects on NK cells [70]. Furthermore, cytokine-inducible levels of different adhesion molecules including VCAM-1 and ICAM-1 are also detectable on MSC which are required for a direct adhesion of MSC to T lymphocytes and subsequent MSC-mediated immunosuppression [71]. Whereas ICAM-1 and VCAM-1 are upregulated in MSC by pro-inflammatory cytokines such as TNFα and IL-1β in combination with γ-IFN, these effects enable MSC to increase adhesion to T cells. Moreover, this cell adhesion-mediated immunosuppression by MSC-to-lymphocyte attachment can be significantly reversed *in vitro* and *in vivo* after inhibition of adhesion via specific blocking antibodies or via adhesion molecule gene knockout [71]. These effects underscore the necessity of a direct MSC-to-cell interaction to convey immune suppression (Figure1, Figure2).

In addition to the MSC’s adhesive capacity by interactive adhesion molecules, further immunosuppressive mechanisms are relayed in part by a variety of different cytokines and other soluble factors which are produced and released by hMSC. These include cytokine receptor ligands (CXCL1, CXCL2, CXCL6), interleukins (IL-1, IL-6, IL-8, IL-10, IL-11, IL-12, IL-14, IL-15), hematopoiesis-stimulating factors (SCF, HGF, G-CSF, M-CSF, GM-CSF), angiogenic growth factors (VEGF, VEGF-D, PlGF), and tropic growth factors (bFGF, FGF-4, EGF, HB-EGF, IGF-I, IGF-II, different forms of PDGF, TGF-β, TGF-β2, TGF-β3) [72-82]. Moreover, expression of nitric oxide synthase by MSC which can be enhanced by TNFα and γ-IFN, is associated with increased production of nitric oxide by MSC and inhibits T cell proliferation [83]. Furthermore, cyclooxygenase as well as indoleamine 2,3-dioxygenase (IDO) which are inducible by γ-IFN, represent synergistically acting key molecules involved in T cell immune suppression. Cyclooxygenase metabolizes polyunsaturated fatty acids like arachidonic acid into prostaglandins such as PGE_2_[84,85] which is involved at least in part in the inhibition of cytokine release by T cells [83]. The molecular mechanism of IDO is suggested to act through the catalyzed conversion of tryptophan to kynurenine, whereby the tryptophan depletion as well as the availability of kynurenine and further metabolites within the hMSC microenvironment contribute to suppress T cell proliferation [86,87] (Figure1, Figure2).

Besides the various immune-modulatory effects of MSC depending on the activation status and the microenvironmental stimulation, there are also controversial data regarding the interaction between MSC and B cells. Studies in a mouse system have documented, that co-culture of BM-MSC with B cells reduced the proliferative capacity and the immunoglobulin (Ig) production of the previously LPS-stimulated immune cells [88]. Moreover, B-cell proliferation was also inhibited and IgM, IgG, and IgA production significantly reduced by BM-hMSC [89]. In contrast, other work has demonstrated an increased proliferation and IgG production of B cells after co-culturing with BM-MSC [90]. Likewise, purified B cells which required cell-to-cell contact with MSC for their activation, exhibited increased polyclonal proliferation and differentiation of naïve and transitional B cells into Ig-producing cells [91].

Although precise molecular interactions still remain unclear, how the two different actions of MSC mediate immune-modulation, either by direct MSC-to-immune cell attachment (e.g. via ICAM-1 and VCAM-1) or by the release of a large panel of cytokines and micro vesicle-containing miRs, it becomes more and more conceivable that both mechanisms may be mutually combined rather than representing exclusive phenomena. Based on this hypothesis, the direct and timely limited MSC-to-immune cell attachment could trigger and enhance a short-term release of required factors which contribute to immune cell-specific (either macrophages, T-, B-, NK- or dendritic cells) responses. In addition, the MSC-immune cell attachment establishes a close interpopulation complex which provides a minimized intercellular space for maximal action of the released cytokines and micro vesicles within this microenvironment (Figure2).

### Clinical use of MSC immune-privileged properties

Besides their immune-modulatory capacity, hMSC also possess immune-privileged properties which means that these cells do not challenge a response of allogeneic immune cells suggesting an active self-protection [92]. Due to low expression levels of MHCI and MHCII antigens, hMSC represent valuable prerequisites for the medicinal use during cell or tissue transplantation. Therefore, the significantly reduced immunogenicity favors a low rejection rate of transplantable hMSC providing a preferred tool in regenerative medicine. However, the hMSC state of immune privilege remains unstable in the course of higher γ-IFN concentrations or differentiation-inducing effects within the hMSC microenvironment which elevate MHCI and particularly MHCII expression and cause a transition from an immune-privileged to an immunogenic phenotype associated with the induction of cellular cytotoxicity or immune rejection [69,93].

Taken together, hMSC represent a heterogeneous population of stromal/stem cells originating from perivascular niches of nearly all tissues which exhibit altered proliferation and differentiation capacities, and provide distinct levels of immune-modulatory activities [92]. This immune-suppressive potential of MSC may not only allow their use for autologous, but also for allogeneic cell therapies in regenerative medicine. Moreover, MSC also display various roles in stem cell homing and tissue repair connected with cell differentiation, cell replacement, and neo-vascularisation. Although a variety of MSC functions still remain controversial and poorly understood, this stem cell population provides a very promising cellular tool in the field of regenerative medicine. The advantage of distinct hMSC, particularly the ethically non-controversial populations derived from neonatal tissues and their clinical potential are useful prerequisites for transplantation in view of cell and/or tissue replacement in combination with their functional biodiversity [94]. Accordingly, MSC have been successfully applied in a variety of clinical trials, e.g. large bone defects [95] and cartilage lesions [96], spinal cord injuries [97], cardiovascular diseases [98], hematological pathologies [99], osteogenesis imperfecta [100], and graft-versus host disease [101]. However, MSC also display a large panel of interactions with diseased cells including a variety of tumor cells which may not always be beneficial for maintaining a healthy microenvironment.

## Interaction of MSC with tumor cells and a tumorigenic microenvironment

According to the heterogeneity of MSC functions already observed during MSC interaction with normal cells and particularly immune cells, there are also a variety of controversial reports suggesting both, tumor-inhibitory and tumor-promoting activities of MSC. These conflicting observations may be related in part to the cellular microenvironment whereby hypoxia establishes an extracellular milieu to reduce aging and enrich poorly differentiated tumor cells and mesenchymal stromal/stem cells. Such an undifferentiated hypoxic microenvironment provides essential cellular interactions and environmental signals for the preferential maintenance of MSC and cancer cells [24,48,49,102].

The contradictory reports on cellular interactions between MSC and tumors can also be associated with different activation levels of MSC which are switched by intracellular triggers such as different miRs. Indeed, different states of MSC activation are relayed by variable expression levels of certain miR including miR-335. High levels of miR-335 expression contribute to a potentially non-activated (silenced) MSC state of auto-maintenance and low amounts of miR-335 suggest an activated state leading to proliferation, migration and differentiation in MSC [103] (Figure1).

### Tumor-promoting effects of MSC by exosomes and MSC-to-tumor cell contacts

Of interest, a functional role in the regulation of a neoplastic development and the formation of metastases has also been attributed to miRs in breast cancer cells, particularly miR-21 and miR-205 for tumor development and miR-126 and miR-335 for breast cancer metastases. Whereas different amounts of miR-335 determine the activation status of MSC, certain expression levels of this miR also contribute to metastasis of breast cancer cells. The constitutive expression of miR-335 targets more than 62 genes [103] including the transcription factor SOX4 and the extracellular matrix component tenascin C. Thus, a down-modulation of this miR and consequently, increased expression of target genes is associated with enhanced risk of mammary tumor spreading and formation of metastasis. Likewise, miR-126 down-modulation is accompanied by an overall tumor growth and proliferation [104]. Therefore, constitutive intracellular amounts of these miRs are considered to exhibit metastasis-inhibitory capabilities and consequently, tumor-inhibitory function may be related to relevant expression levels of additional miRs.

Tumor-promoting effects are also attributed to an epithelial-to-mesenchymal transition (EMT), whereby MSC play a crucial role. Thus, EMT represents a key event for colorectal cancer progression and MSC are implicated in this process [105]. Likewise, EMT is involved in metastatic breast cancer. During conversion of epithelial breast cancer cells to a mesenchymal phenotype and loss of apical-basolateral polarity, the tumor cells acquire morphological features and stem cell-like characteristics as compared to MSC [106] and may therefore resemble breast cancer stem cells. In addition, MSC-released exosomes can induce vascular endothelial growth factor (VEGF) expression in tumor cells by activating extracellular signal-regulated kinase1/2 (ERK1/2) pathway [107] which contributes to enhanced angiogenesis and tumor growth.

Interactions of MSC with breast cancer cells include the formation of gap junctions [108] The gap junction represents a membrane spanning channel (connexon) on each of the junctional cell types formed by a hexamer of connexins and allows gap junctional intercellular communication (GJIC) by transcellular exchange of low molecular weight compounds including calcium ions (Ca^2+^), inositol phosphates (different IP_3_ and IP_4_) and cyclic nucleotides Thus, a derailed GJIC or defects in GJIC can modulate the metastatic potential of the breast cancer cells [109]. Moreover, loss of GJIC correlates with malignant phenotype progression in neoplastic mammary tissue [110].

Consequently, MSC provide direct cell-cell contact with tumor cells whereby these interactions rather than soluble factors predominantly promote tumor cell proliferation and survival *in vitro*[111]. Indeed, cellular interactions between MSC and tumor cells are facilitated via ICAM-1 and VCAM-1 receptors which also play a role in normal tissues during MSC and T cell interactions. Correlating to the presence of ICAM-1 on the MSC, breast cancer cells express cell surface-associated mucin 1 (MUC1). MUC1 motifs can interact with ICAM-1 molecules and such ICAM-1-MUC1 interactions initiate an actin-based cell invasive motility [112]. MUC1 (CD227, DF3, CA15-3) represents a transmembrane heterodimeric glycoprotein normally expressed at the apical surface of mammary epithelium [113]. However, during breast cancer progression, cell polarization is frequently lost and consequently, MUC1 circumferentially distributes around the entire plasma membrane of the breast cancer cell or is highly overexpressed throughout the cytosol [114] (Figure2).

At the molecular level, ICAM-1-MUC1 interactions activate Src kinase and subsequent Src-mediated phosphorylation of the cytoplasmic domain of MUC1 at two highly conserved Y-x-x-P sequences (Y35VPP38 and Y60TNP63) attribute to a direct recruitment of CrkL binding via its SH2 domain. The SH2/SH3 adaptor protein CrkL is involved in the regulation of cell migration through the association with guanine nucleotide exchange factors such as Dock180 to catalyze GTPase activation and GDP/GTP exchange. Consequently, the ICAM-1-MUC1-mediated Src-CrkL complex formation further activates the small GTPases Rac1 and Cdc42 which are key effectors of the actin cytoskeleton protrusion machinery and thereby, reorganize the actin cytoskeleton and promote an actin-based cell motility [112]. Supportive studies have demonstrated, that MSC contribute to alter stiffness via small GTPases Rho A and Cdc42 [115]. Finally, the enhanced motility of the MUC1-carrying tumor cells contributes to a significantly elevated metastatic potential.

### Tumor-inhibitory effects of MSC by ECM and soluble factors

Together, these studies suggest that direct cell-to-cell interactions between MSC and tumor cells via gap junctions and the exchange of exosomes or via membrane receptor assembly promote tumor cell growth. In contrast, tumor inhibitory effects of MSC are reported preferentially via the ECM compounds of the microenvironment and soluble factors (Figure2). Thus, extracts from Wharton’s jelly-derived MSC – either cell lysates or cell supernatants - inhibit cell growth of a variety of cancer cell lines including breast cancer, ovarian carcinoma, and osteosarcoma cells [116]. Moreover, β-IFN secreted by MSC within the tumor microenvironment and intratumorally produced β-IFN significantly reduces breast cancer growth and metastatic potential by inactivation of the constitutive phosphorylation of signal transducer activator transcription factor 3 (Stat3) via Src, and Akt kinases and a concomitant down-regulation of c-Myc and MMP2 expression [117]. Finally, ECM components produced by MSC contribute to arrest the growth of metastatic tumor cells by an up-regulation of the tumor suppressor phosphatase and tensin homolog (PTEN) in the tumor cells [98]. In addition, dickkopf (DKK1)-producing MSC are capable of inhibiting cancer cell proliferation independent of the tumor-inhibitory effect of MSC-generated ECM compounds [118].

More detailed studies in a tumor model revealed that UC-MSC possess a significantly elevated migratory potential as compared to BM-MSC. The capacity to migrate towards glioma cells is accompanied by an elevated expression of IL-8 receptors and CXC chemokine receptors-1 and −2 on UC-MSC [119]. Malignant glioma cells have been demonstrated to recruit MSC by secreting angiogenic factors, such as VEGF [120], and cytokines, including IL-8 [121]. In addition, MMPs and the corresponding MMP tissue inhibitors are associated with MSC migration to injured tissues [122]. *In vivo* application of MSC into the contralateral hemisphere of a glioma in rats was associated with migration of the MSC towards the tumor through the corpus callosum. Moreover, intratumoral injection of MSC resulted in a significant inhibition of tumor growth and increased the survival of these rats [123]. In the human system, MSC integrate into gliomas after intravascular or local injection [124]. Since MSC are capable of homing to gliomas (likewise to other tumor entities), these multipotent stem cells have been suggested a powerful cellular vehicle to target tumor cells [125]. In addition, the capability of MSC to release exosomes, represents a potentially useful tool for a specific delivery of customized anti-cancer compounds [126,127].

In conclusion, these effects suggest that MSC display tumor-inhibitory functions preferably via the ECM and the release of distinct factors into the tumor surrounding microenvironment, whereas tumor-promoting effects are relayed predominantly by direct cell-to-cell interaction and the exchange of membrane vesicles.

### Aberrant MSC

**Figure 3:**
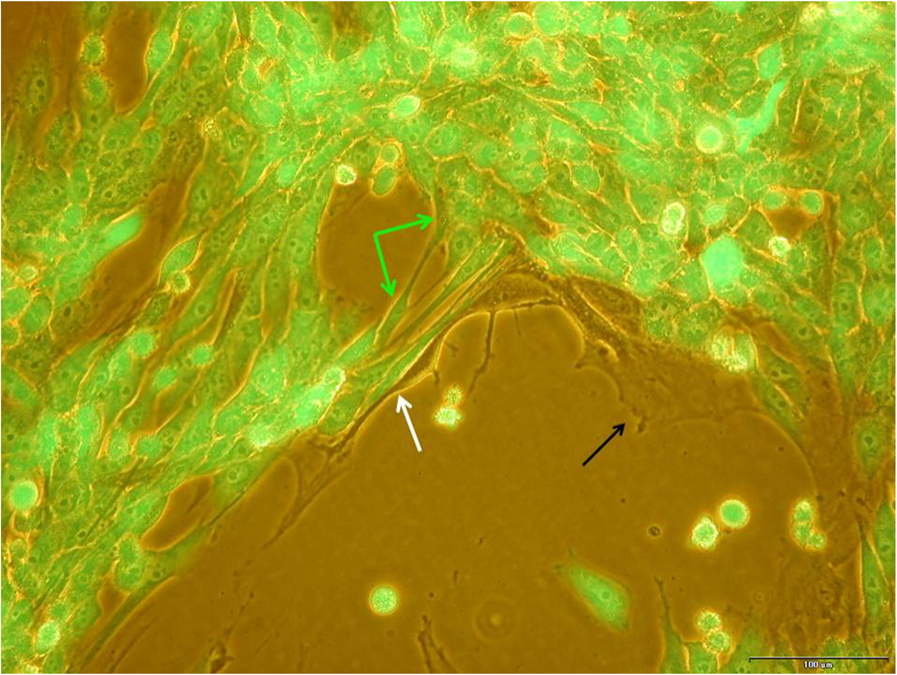
**Phase contrast/fluorescence microscopy overlay of primary umbilical cord-derived hMSC (black arrow) and lentivirus vector GFP- transfected MDA-MB-231 human breast carcinoma cells (green cells) originally seeded in a ratio of 60:40 (MSC: MDA-MB-231) and co-cultured for 7d.** A certain amount of spindle-shaped MSC surrounded the tumor cells (white arrow). However, as judged by the morphology, a small amount of these spindle-shaped cells appeared with green fluorescence (green arrows) which may indicate a possible fusion of MSC with GFP-containing exosomes or a complete MSC-breast cancer cell fusion with 2 nuclei (green arrows). Bar graph demonstrates 100 μm.

A variety of further evidences suggest that disease- and cancer-associated stromal cells are distinctly abnormal as compared to MSC from healthy tissues. E.g., multiple myeloma represents a plasma cell malignancy characterized by complex heterogeneous cytogenetic abnormalities and a significant amount of MSC derived from multiple myeloma carry chromosomal abnormalities and defects in gene and protein expression [128]. Likewise, MSC cultured from patients with severe systemic sclerosis constitutively release higher levels of cytokines/chemokines such as SDF-1 and VEGF and stimulate microvascular endothelial cell angiogenesis much more efficiently as compared to normal BM-MSC [129]. The development of certain sarcomas as mesenchymal tumors has also been attributed to originate from aberrant MSC, whereby the WNT signaling pathway plays an important role to exert tumor suppression and conversely, its inhibition is accompanied by sarcoma genesis [130]. Moreover, growth of BM-hMSC in long-term cultures (5–106 weeks) was associated with a spontaneous malignant transformation which occurred in about half of these cultures and demonstrated a significantly increased proliferation rate and an altered morphology and phenotype [131]. The close vicinity of MSC to neighboring cells via adhesion receptors and/or the exchange of exosomes can also favor cell fusion and the formation of multinucleated cells which alters MSC functionality (Figure1, Figure2). Previous observations with BM-hMSC in certain neurodegenerative disorders and flow cytometry of fluorescently-labeled MSC in co-culture with nucleus pulposus cells revealed evidence for the formation of gap junctions and some cell fusion although these phenomena occur at low frequency [132,133]. Similar observations are confirmed during co-culture of UC-hMSC with the human breast cancer cell line MDA-MB-231 (Figure3). 

The close vicinity of MSC to the tumor entity also enables interactions with biological material obtained from necrotic tumor tissue and dying cancer cells. Such necrotic factors – also termed damage-associated molecular patterns (DAMPs), can activate MSC by enhancing their angiogenic capacity or affect the immune-regulatory potential of MSC by interfering with the expression of indoleamine 2,3-dioxygenase in MSC [134]. Moreover, whole necrotic vesicles or apoptotic bodies from dead tumor cells can be incorporated into MSC by engulfment or fusion contributing to transform the MSC functionality. Thus, BM-hMSC which have been cultured in tumor-conditioned medium for up to 30d acquire functional properties of carcinoma-associated fibroblasts (CAF). These CAF-like transformed MSC express myofibroblast markers, including α-smooth muscle actin and fibroblast surface protein and produce sustained levels of SDF-1 (Figure1, Figure2). Simultaneously, these CAF-like MSC also promote tumor cell growth both, *in vitro* and in an *in vivo* co-implantation model [135]. Likewise, MSC transformed with specific mutations play an important role in sarcoma genesis and in the control of sarcoma tumor growth [136,137]. Furthermore, MSC which have been isolated from primary breast cancer tissues significantly promote tumor growth and size following co-transplantation with breast cancer cells. These tumor-derived MSC release EGF and promote mammosphere formation via the PI3K/Akt signaling pathway [138]. Finally, MSC display some similarities to aged normal mammary epithelial cells and tumorigenic human breast epithelial cells particularly after EMT with respect to the gene expression pattern [139] and some surface receptor levels [140,141]. This functional association underscores the involvement of stem cells associated with breast cancer and their supportive role according to stimulatory effects from the microenvironment [142,143].

Together, MSC can directly interact with a variety of different adjacent normal cell types either via adhesion molecules and gap junctions or via exchange of a large panel of cytokines/chemokines, exosome-containing miRs and ECM components. In case of tumor cells, such interactions with MSC result in the support of tumor-promoting and tumor-inhibiting activities, respectively. Moreover, uptake of DAMPs and fusion with vesicles or apoptotic bodies from tumor cells contribute to alter the MSC functionality into an aberrant phenotype.

## Conclusions

MSC demonstrate a functional heterogeneity within different tissue origins. This heterogeneity is even displayed within the same population and suggests MSC subpopulations exhibiting specialized functions according to local tissue requirements which may develop from certain precursor cells and still maintain stem cell properties [144]. Thereby, MSC can maturate predominantly along phenotypes of the mesoderm lineage, protect hematopoietic stem cells, modulate the reactivity of immune cells (e.g. macrophages, NK-, T-, B-, dendritic cells), activate endothelial cells and either suppress tumor growth via ECM and soluble factors (e.g. gliomas) or enhance the motility and metastatic capacity of tumor cells via exosome exchange and direct cell-to-cell contact (e.g. breast cancer cells). The heterogeneity of MSC is also reflected by molecular triggers such as cytokines or the intracellular expression levels of certain miRs that alter the MSC state of activation in order to self-renew, maintain stemness, regulate stem cell homeostasis, undergo differentiation, migrate towards tissue damage, modulate immune cell functions, support angiogenesis, and promote or inhibit cancer growth and metastasis.

Whereas MSC appear to function almost as a cellular all-rounder in support of tissue homeostasis and plasticity, these unique capabilities of biodiversity may also be misused during transformation into aberrant MSC phenotypes.

## Competing interests

The authors declare that they have no competing interests.

## Authors contribution

AO contributed to Figure1 and Figure2. RH. finalized the figures and drafted the manuscript. All authors read and approved the final manuscript.
